# Integrated analysis of racial disparities in genomic architecture identifies a trans‐ancestry prognostic subtype in bladder cancer

**DOI:** 10.1002/1878-0261.13360

**Published:** 2022-12-29

**Authors:** Baifeng Zhang, Peilin Jia, Jiayin Wang, Guangsheng Pei, Changxi Wang, Shimei Pei, Xiangchun Li, Zhongming Zhao, Xin Yi, Xin‐yuan Guan, Yi Huang

**Affiliations:** ^1^ Departments of Clinical Oncology The University of Hong Kong‐Shenzhen Hospital China; ^2^ Departments of Clinical Oncology, Li Ka Shing Faculty of Medicine The University of Hong Kong China; ^3^ Geneplus‐Beijing China; ^4^ Center for Precision Health, School of Biomedical Informatics The University of Texas Health Science Center at Houston TX USA; ^5^ Department of Computer Science and Technology, School of Electronic and Information Engineering Xi'an Jiaotong University Shaanxi China; ^6^ Department of Epidemiology and Biostatistics Tianjin Medical University Cancer Institute and Hospital China; ^7^ Luohu people's hospital Shenzhen China

**Keywords:** bladder cancer, genomic architectures, tumor heterogeneity, tumor microenvironment

## Abstract

The incidence of bladder cancer and patient survival vary greatly among different populations, but the influence of the associated molecular features and evolutionary processes on its clinical treatment and prognostication remains unknown. Here, we analyze the genomic architectures of 505 bladder cancer patients from Asian/Black/White populations. We identify a previously unknown association between *AHNAK* mutations and activity of the APOBEC‐a mutational signature, the activity of which varied substantially across populations. All significantly mutated genes but only half of arm‐level somatic copy number alterations (SCNAs) are enriched with clonal events, indicating large‐scale SCNAs as rich sources of bladder cancer clonal diversities. The prevalence of *TP53* and *ATM* clonal mutations as well as the associated burden of SCNAs is significantly higher in Whites/Blacks than in Asians. We identify a trans‐ancestry prognostic subtype of bladder cancer characterized by enrichment of non‐muscle‐invasive patients and muscle‐invasive patients with good prognosis, increased *CREBBP/FGFR3/HRAS/NFE2L2* mutations, decreased intra‐tumor heterogeneity and genome instability, and an activated tumor microenvironment.

## Graphical abstract

Our study demonstrates the presence of great racial disparities in the genomic architecture of bladder cancer and identifies a trans‐ancestry prognostic subtype with distinct genomic features and good clinical outcome. These findings may be useful for guiding personalized treatment for bladder cancer.

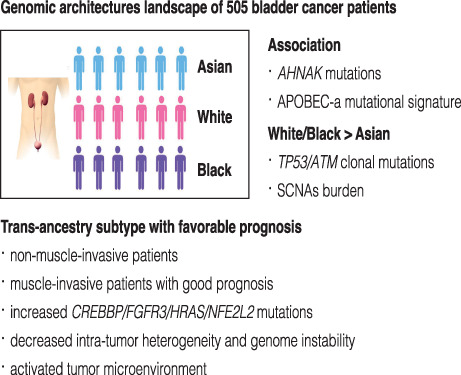

**Abbreviations**
APOBEC: apolipoprotein B mRNA editing enzyme
BLCA: bladder cancer
CCFs: cancer cell fractions
COSMIC: Catalogue of Somatic Mutations in Cancer
GDC: the genomic data commons
MAD: the median absolute deviation
MIBC: muscle‐invasive bladder cancer
MMR: mismatch repair deficient
NER: nucleotide excision repair
NMF: non‐negative matrix factorization
NMIBC: non‐muscle‐invasive bladder cancer
SCNAs: somatic copy number alterations
SMGs: significantly mutated genes
SNVs: single nucleotide variants
ssGSEA: single sample gene set enrichment analysis
TCGA: The Cancer Genome Atlas
TH17: T helper 17 cells
TH2: T helper 2 cells
WGD: whole genome doubling

## 1 Introduction

Bladder cancer is the sixth most common type of adult cancer with almost 430 000 new cases diagnosed per year [1]. Bladder cancer is classified into non‐muscle‐invasive bladder cancer (NMIBC) and muscle‐invasive bladder cancer (MIBC) according to their histopathological features and clinical behaviors. NMIBC, the major subtype of bladder cancer that accounts for 70–80% of all newly diagnosed cases, has better overall survival but high recurrence rate of 31–78% while the rest of patients with muscle‐invasive or metastatic diseases exhibit quite low survival rate [2, 3]. The genetic or molecular basis underlying such great disparities in clinical outcomes between NMIBC and MIBC remains largely unclear.

NMIBC and MIBC are supposed to develop through different evolutionary paths on distinct genetic background. The genetic architecture of MIBC has become increasingly clear with the accomplishment of several large‐scale genome sequencing projects in large datasets of MIBC patients [4, 5, 6]. Characterization of the genetic causes of NMIBC by large‐scale approaches had only been performed in a relatively small number of patients [7, 8]. These studies identified a number of new driver events, such as truncating mutations of the chromatin remodeling genes (*UTX*, *MLL*‐*MLL3*, *ARID1A*, and others) and recurrent fusions involving *FGFR3* and *TACC3*, in addition to the well‐known TP53‐RB1 and FGFR3‐Ras pathways that are mutated frequently in MIBC and NMIBC, respectively [7, 8]. Bladder cancer is a heterogeneous group of diseases with distinct subtypes exhibiting different mutational signatures and expression features [4]. However, these studies primarily focused on the somatic events with elevated mutation frequencies among different patients or subtypes [5, 6, 7, 8].

The degrees of intra‐ and inter‐tumor heterogeneities can be assessed by analyzing the genomic architectures of individual bladder cancer patients. One previous study analyzing the clonality of MIBC in a small number of pre‐ and post‐chemotherapy samples revealed that the extensive and dynamic clonal evolution found in MIBC patients may lead to chemotherapy resistance [9]. It was expected that the genomic architectures would have great influence on the clinical outcomes of bladder cancer and somatic driver events acquired at different evolutionary stages would also have different prognostic values. However, few of the genetic prognostic biomarkers for bladder cancer had been developed utilizing their clonal or subclonal states in large datasets of patients with different genetic background, despite the well‐documented disparities in bladder cancer risk and survival across races/ethnics in epidemiological studies [10]. Whites are more than twice likely to develop bladder cancer than Blacks while Asian Americans have lower risk of developing bladder cancer than Whites and Blacks [11]. The clinical outcomes of bladder cancer also exhibit racial disparities with shorter survival observed in Blacks [10]. Moreover, these previous prognostic studies also focused only on single level of molecular data and no study had characterized the overall genomic architectures of individual bladder cancer patients based on both large‐scale somatic copy number alterations (SCNAs) and single nucleotide variants (SNVs) simultaneously to stratify distinct prognostic subtypes. In the study, we tried to investigate that with disparities in genetic background, life history, and mutagens exposure, whether bladder cancer patients from different populations might show different mutational signatures and therefore further lead to discrepancies in mutational features and genomic architectures.

To more comprehensively understand the genetic diversities of bladder cancer, we collect large‐scale genomic data from The Cancer Genome Atlas (Dataset 1: TCGA‐BLCA, *N* = 408) [4, 6] and the Chinese population (Dataset 2: Chinese‐BLCA, *N* = 97) [7, 8]. Dataset 1 consists of MIBC patients from three different racial populations (324 White, 23 Black, 43 Asian and 18 not reported) and Dataset 2 consists of both MIBC (60 Asian) and NMIBC (37 Asian) patients from Asia only. We use the MIBC or NMBIC patients from Dataset 2 separately or jointly to test or validate some of the findings observed in Dataset 1. In total, our study datasets include 468 MIBC and 37 NMIBC patients. We compare the differences in mutational signatures among Asian, Black and White populations and further characterize the association between driver gene mutations and the activities of mutational signatures. We perform prognostic analysis of MIBC in Dataset 1 patients with survival information available and further cluster the MIBC and NMBIC patients according to their molecular patterns. After quantifying the cancer cell fractions (CCFs) of all potential prognostic somatic events, we categorize the bladder cancer patients into distinct prognostic subtypes showing differences in intratumor heterogeneity, genome instability, metastatic ability, and immune features.

## 2 Materials and methods

### 2.1 Data sources and sample information

Raw whole‐exome sequencing, RNA‐seq data, and clinical information on the Chinese bladder cancer patients were downloaded from the Sequence Read Archive (SRA063495) [7]. Whole‐exome sequencing bam files, gene expression read counts, and clinical information in the TCGA‐BLCA dataset were downloaded from the Genomic Data Commons (GDC) data portal (http://gdc‐portal.nci.nih.gov). All bladder cancer patients were classified into the NMIBC or MIBC subtype based on their TNM stages. Patients who had metastatic diseases at the time of biopsy or within 1 year after biopsy were identified as metastatic and patients who were followed up for at least 1 year and did not show metastatic diseases during the entire follow‐up period were identified as non‐metastatic. Data on WGD were downloaded from the Genomic Data Commons (GDC) website (https://gdc.cancer.gov/about‐data/publications/panimmune) [12]. All bladder cancer patients were classified into Asian, Black, and White populations based on their racial origins (Tables S1 and S2).

### 2.2 Identification of SNVs and SCNAs

Bam files for the tumor and matched normal samples were processed as input. Somatic mutations were called by using mutect2 software (version 4.1.4) [13] and further filtered with a read depth of at least 10× in the germline and tumor samples, a maximum of two variant supporting reads in the germline, a minimum tumor variant allele frequency of 10%, and a maximum germline variant allele frequency of 2%. Allelic SCNAs were called with the ReCapSeg module in GATK following the Broad's GATK documentation (https://gatk.broadinstitute.org/hc/en‐us/articles/360035531092).

### 2.3 Estimation of intratumor genetic heterogeneity

To estimate the level of intratumor genetic heterogeneity in bladder cancer, we calculated each tumor's MATH value from the median absolute deviation (MAD) and the median of its mutant allele fractions at somatically mutated loci: MATH = 100 * MAD/median [14].

### 2.4 RNA‐seq analysis and estimation of immune cell score

Bam files for paired samples were processed to quantify the transcript abundance levels by using kallisto (version 0.43) [15]. We then used tximport (version 1.4.0) [16] r package to convert transcript level value into gene level. We used signature genes from previous study [17] to infer the infiltration levels of different types of immune cells using ssGSEA [18] algorithm. Normalized RNA‐seq datasets were provided as the inputs for the execution with ‘gsva (data, list of signatures, method = ‘ssgsea’) [19].

### 2.5 Mutation significance analysis

mutsig2cv algorithm (version 3.11) [20, 21] was used to identify the SMGs which were further filtered with *P* < 0.05 & *q* < 0.1. An SMG should express in above 75% of the TCGA‐BLCA patients and with at least three read counts. Also, they should be reported as the census of human cancer genes (https://cancer.sanger.ac.uk/census, version: 4_08_47_12_2018).

### 2.6 Mutation clonality analysis

We used the updated version of absolutev1.2 algorithm (version 1.2) [22, 23] with allelic copy number and mutation data as inputs to infer the ploidies and CCFs of tumor cells. SNVs and SCNAs were defined as clonal if the probabilities of observing CCFs ≥ 0.95 were greater than 0.5 (Pr_(CCF ≥ 0.95)_ > 0.5) or subclonal otherwise [24, 25]. To assess whether a specific gene or arm level event was enriched with clonal or subclonal mutations, we used permutation tests repeated by 10 000 times to obtain a *P* value of clonal enrichment by dividing the times when the observed clonal/subclonal ratio was greater than the expected ratio by 10 000. We applied the method of constructing the potential temporal order of mutation acquisitions during tumor evolution [26].

### 2.7 Mutational signature analysis

We applied a Bayesian variant of NMF (BayesNMF) with the functions of optimal inference for the number of signatures and *de novo* signature discovery to identify the mutational signatures [27]. Comparison of the signatures extracted from three populations (Asian, Black, and White) and 30 COSMIC signatures was performed using the standard hierarchical clustering r package with a distance measurement of ‘cosine’ similarity or ‘Pearson’ correlation. The MSig clustering analysis was performed using a standard hierarchical clustering in r, with a ‘Euclidian’ distance for the signature activity matrix and a ‘ward.D' linkage tree. By using the Elbow method to look at the total within‐cluster sum of square (wss) as a function of the number of clusters, the number of MSig clusters (*K* = 4) was chosen so that adding another cluster does not improve much better the total wss.

### 2.8 Signature enrichment analysis

To search for genes whose mutation statuses were associated with the activity of a specific signature, we applied a permutation statistical test [27] to compare, for each gene, the signature activities between samples with and without mutations in the gene. Firstly, to remove the inflation in the number of mutations in each gene associated with the elevated background mutation rates, we randomly produced a total of 10^4^ permutated mutation matrix in which the total counts of gene‐specific and sample‐specific mutations were kept the same as those observed in our patient datasets, following an approach described by Strona et al. [28]. We used the one‐tailed Wilcoxon rank‐sum *P* value to compare the signature activity between mutant and wild‐type samples of a specific gene. To get the final *P* value for a given gene, we calculated the fraction of permutated matrix with a test *P* value equal to or more extreme than the observed matrix (Sum of *P*_observed_ ≥ *P*_random_ times/the total number of permutations). To increase the computational efficiency, we analyzed only 174 genes with a non‐silent mutation frequency of 5% or above, 26 SMGs, the APOBEC family member gene sets, and the human DNA repair gene sets that had been reported to be associated with some mutational signature activities (downloaded from https://www.mdanderson.org/documents/Labs/Wood‐Laboratory/human‐dna‐repair‐genes.html). We corrected for multiple hypothesis testing using the Benjamini–Hochberg procedure and used FDR *q* < 0.1 as the significance threshold.

### 2.9 Calculation of the SCNA scores

We calculated the SCNA scores according to a previous study with minor modifications [29]. Our calculation of SCNA was based on the integer allelic copy number calls generated by absolute (version 1.2) [22] which took the tumor purities into consideration. The absolute copy number of each segment was determined and a chromosomal arm event would be called out if the cumulative length of the deletions (genomic segments with minor alleles of 0 copy) or amplifications (genomic segments with major alleles of two or more copies) on this arm was > 50% of the chromosome arm. Chromosome level events were distinguished from arm level events when both arms of a chromosome had the same copy number changes (in sign). After excluding the arm‐ and chromosome‐level copy number changes, the remaining segments with SCNAs were defined as focal changes. Based on the copy number change sign associated with each segment *x* of a chromosome, arm, and focal event *t*, the assigned score *S* were defined as:

$$\mathrm{Sx}=\left\{\begin{array}{c} +2\;\mathrm{one}\;\text{allele}\;\mathrm{had}\;\text{amplification and the}\\ \mathrm{another}\;\mathrm{one}\;\mathrm{had}\;\text{deletion}\\ +1\;\text{both the major and minor alleles}\;\mathrm{had}\\ \;\text{amplifications or deletions}\\ +1\;\mathrm{one}\;\text{of major and minor allele was in}\\ \;\text{normal state}\\ 0\;\text{both major and minor alleles were in}\\ \text{normal state} \end{array}\right..$$

The sum score of deletions or amplifications at the chromosome (*ChromL*), arm (*ArmL*), and focal levels (*FocalL*) was calculated separately:

$$\text{ChromL}=\sum_{t\;\varepsilon \;\text{Chrom}}\mathrm{Sx}$$

$$\;\text{ArmL}=\;\sum_{t\;\varepsilon \;\mathrm{Arm}}\mathrm{Sx}$$

$$\text{FocalL}=\sum_{t\;\varepsilon \;\text{Focal}}\mathrm{Sx}$$

The *ChromL*, *ArmL*, and *FocalL* scores of each tumor sample were normalized to the mean and standard deviation calculated among all samples. In addition, we divided the summed score of all SCNA segments by the total length of genomic segments to get ‘total normalized SCNA level’ score for each tumor sample, which represented the integrated level of SCNAs among the genome [29].

### 2.10 Unsupervised clustering based on genomic architecture

We used the CCFs of 26 SMGs and frequent arm level SCNAs to create a numerical matrix (samples as columns) and then applied the NMF ‘lee’ algorithm to perform molecular subtyping of bladder cancer. After manual inspection, we chose the *K* = 2 solution and reported two clusters (clusters A vs. B).

### 2.11 Statistical analysis

Two‐sided Mann–Whitney and Fisher's exact tests were performed with the r functions Wilcox.test and chisq.test to generate the empirical *P* values, respectively. *P* values were adjusted for multiple hypothesis tests using the r function p.adjust with the ‘fdr’ option.

### 2.12 Survival analysis

Asian bladder cancer patients from Asia dataset were excluded in survival analysis due to the lack of some key clinical information. Chi‐square test statistics in Kaplan–Meier curves were computed using log‐rank tests. *P* values were also calculated from multivariate Cox proportional hazards regression models using the r package ‘survival’.

## 3 Results

### 3.1 Racial differences in mutational signatures

To dissect the somatic mutation landscapes of the 505 bladder cancer patients in our study, mutect2 [13] and recapseg [30] were used to call somatic mutations and allelic SCNAs with the whole‐exome sequencing data. After further filtering steps (Materials and methods), 136 346 somatic mutations [132 100 SNVs and 4246 short insertions/deletions (Indels)] and 21 779 SCNA segments were generated for downstream analysis. We then inferred the CCFs of SNVs and SCNAs simultaneously for each patient. By combining all somatic events from all patients, 11.2% of SNVs, 30.5% of Indels, and 41.1% of SCNAs were identified as subclonal events (Table S3).

**Fig. 1:**
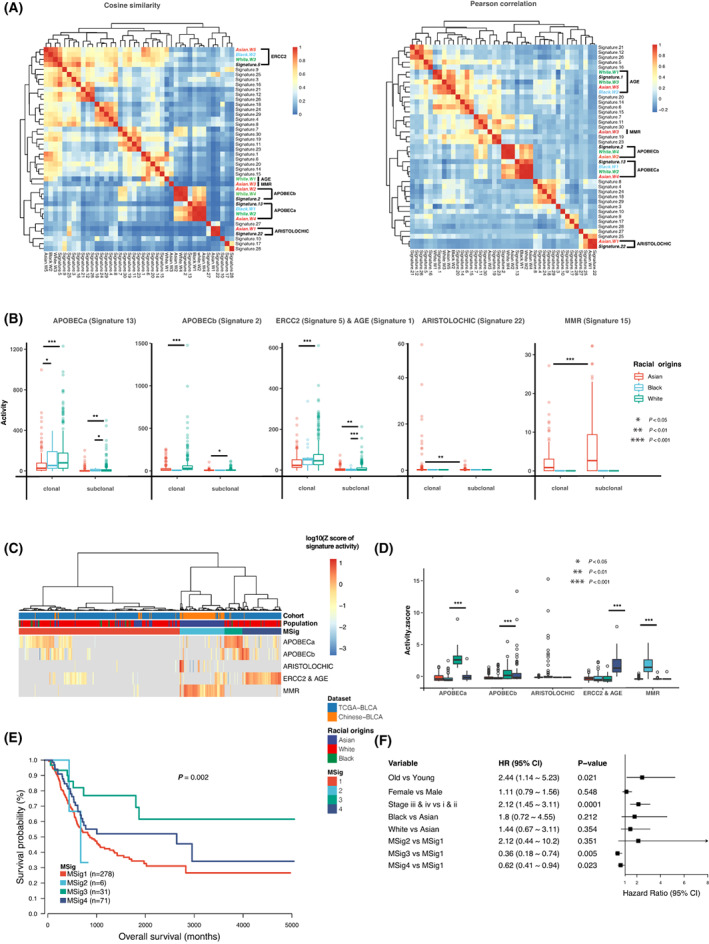
Mutational signatures of bladder cancer among Asian—140, Black—23, and White—324 populations. (A) Unsupervised hierarchical clustering of signatures identified in our datasets and the 30 signatures described by COSMIC (the Catalogue of Somatic Mutations in Cancer). The cosine similarity and Pearson correlation value within signatures are clustered and displayed by heatmap. (B) Differences in the mutational signature activities among three populations in clonal and subclonal levels (red: Asian, blue: Black, green: White). *P* values were calculated using the Wilcoxon rank‐sum test: 0.01 < *P* < 0.05, 0.001 < *P* < 0.01, *P* < 0.001 are shown as *, **, and ***, respectively. Data are plotted as mean ± SEM. (C) Unsupervised hierarchical clustering of 487 samples [MSig (mutational signature) clusters] based on the number of mutations [log_10_(*Z* scores)] assigned to the five mutational processes. Details in *mutational signature analysis* in Materials and methods section. (D) Enriched signature features of the four MSig clusters. *P* values were calculated using the Wilcoxon rank‐sum test: *P* < 0.001 are shown as ***, respectively. Data are plotted as mean ± SEM. (E) Kaplan–Meier survival analysis of the four MSig clusters using only TCGA data due to the lack of some key clinical information in Chinese dataset. Chi‐square test statistics in Kaplan–Meier curves were computed using log‐rank tests. (F) Multivariate cox regression analysis of survival by including MSig clusters, racial origins, age, gender, and TNM stage (using only TCGA data). Data are plotted as mean ± SEM. *P* values were also calculated from multivariate Cox proportional hazards regression models.

The mutational signatures document the evolutionary history of interactions between multiple exogenous and endogenous mutational processes which consequently result in the accumulation of somatic mutations in tumor cells. Bladder cancer patients from different populations may have distinct genetic background, life history, or mutagen exposure. To investigate whether there were any racial differences in mutational signatures during bladder cancer evolution, we first divided our study datasets into three populations of Asian (140), Black (23), and White (324) origins. We extracted the mutational signatures from SNVs with Bayesian non‐negative matrix factorization (NMF). We identified five signatures in Asian, two signatures in Black, and four signatures in White (Fig. 1A and Fig. S1). Through the clustering analysis of the cosine similarities and Pearson correlation values between these signatures and the 30 Sanger signatures with reported roles in mutagenesis [31, 32, 33], the five mutational signatures identified in our datasets could be grouped into categories resembling the APOBEC‐a, APOBEC‐b, ERCC2 & AGE, ARISTOLOCHIC, and MMR Sanger signatures (cosine similarities between 0.68 and 0.97, Fig. 1A, Fig. S2 and Table S4). APOBEC‐a and APOBEC‐b signatures, accounting for the majority of mutations in bladder cancer, appeared to be more likely to occur early during bladder cancer development [4]. The ERCC2 & AGE signature included one signature mediated by *ERCC2* mutations [27] and another signature correlated with the age of cancer diagnosis. ARISTOLOCHIC signature was related to exposures to aristolochic acid. The last mutational signature showed relatively low similarity (cosine similarity of 0.68) to the MMR signature which had not been reported in bladder cancer previously.

Of these five signatures, the activities of both APOBEC‐a and ERCC2 & AGE signatures were the highest in White and the lowest in Asian, regardless of the clonal/subclonal states of mutations (*P* < 0.001 & FDR < 0.1, Fig. 1B). The ARISTOLOCHIC and MMR‐like signatures occurred only in Asian and were both enriched in the subclonal state (*P* < 0.001 & FDR < 0.1, Fig. 1B and Fig. S3). These observations might suggest the presence of population variations in mutational processes operating at different evolution stages during bladder cancer development. To further assess the potential clinical association of these mutational signatures, we performed unsupervised clustering of the patients in Datasets 1 and 2 according to their mutational signature activities and identified four mutational signature clusters (MSig1 to MSig4, Fig. 1C). Different clusters of patients showed varied activities of different mutational signatures (Fig. 1D) and were associated with distinct clinical outcomes (Fig. 1E, *P* = 0.002). Cluster MSig2 showed enrichment in Asian patients (Fig. 1C) and had high MMR‐like signature activity (*P* < 0.001 & FDR < 0.1, Fig. 1D). Prognostic analysis of Dataset 1 revealed that MIBC samples in cluster MSig2 tended to have the shortest overall survival (Fig. 1E) and this observation had to be further investigated in future studies as the number of samples with survival information available in cluster MSig2 was quite small in our study. Patients in the MSig3 and MSig4 clusters had high levels of APOBEC and *ERCC2* & AGE signature mutagenesis (Fig. 1D), respectively. Multivariate Cox regression analysis of Dataset 1 patients showed that those patients in the MSig3 and MSig4 clusters had better survival than those belonging to MSig1 cluster (Fig. 1F).

The Asian dataset in our study was consisted of patients from both Europe/USA areas (Dataset 1: MIBC) and Asia areas (Dataset 2: MIBC and NMIBC) and we compared the relative contribution of the mutational signatures among these three Asian patient groups and found that the activities of APOBEC‐a and ‐b signatures were relatively higher in MIBC patients from Dataset 2 than the other two groups of Asian patients. Both MIBC and NMIBC patients from Dataset 2 showed significantly high levels of MMR‐like signature activities compared with MIBC samples from Dataset 1 (Fig. S3). Nevertheless, more studies and samples were needed to validate the observed racial differences in the activity of MMR‐like signature and we could not rule out the potential influence of study batch effect on this observation due to the following reasons: (a) the MMR‐like signature with minor exposures could only be observed in Dataset 2 generally; (b) most of the mutations contributed to the MMR‐like signature were subclonal; (c) the MMR‐like signature had a relatively low level of similarity to any known COSMIC signatures; and (d) little previous evidence suggested the presence of this signature in bladder cancer (Fig. S4).

### 3.2 Gene mutations potentially associated with signature activities

To explore whether there were any genes whose mutations were associated with the mutational signature activities, we performed signature enrichment analysis for those genes mutated at elevated frequencies with permutation tests which could control the inflation that an increase in the overall mutation burden in each sample or in the total number of mutations in each gene usually correlated with increased signature activity. To determine the significance level of associations between the elevated mutation frequency of each gene and the increased activity of each signature, the mutation burden attributed to a given signature from tumors that harbored non‐silent mutations in the gene was compared with tumors that did not by combining the two study datasets (Materials and methods).

**Fig. 2:**
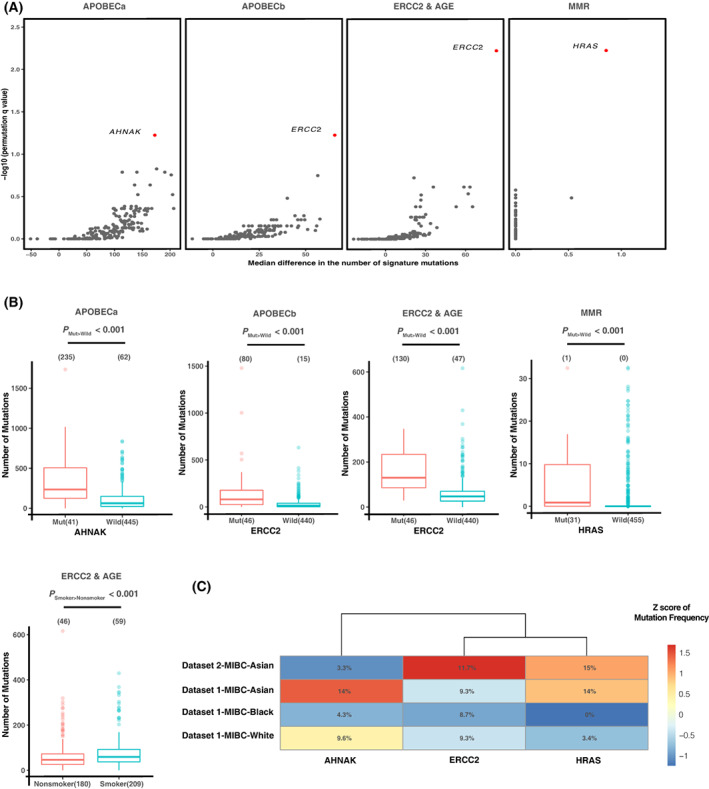
Gene mutations associated with signature activities. (A) Mutation enrichment analysis identifies an association between four somatically mutated genes and signature activities. *X*‐axis: The difference of median mutation burden attributed to a giver signature from tumors that harbored non‐silent mutations in the gene comparing with tumor that did not. *Y*‐axis: The Benjamini–Hochberg corrected *P* value, which calculated by the fraction of 10^4^ permutated matrix with a one‐tailed Wilcoxon rank‐sum test *P* value equal to or more extreme than the observed matrix (sum of *P*_observed_ ≥ *P*_random_ times/the total number of permutations). Details in *signature enrichment analysis* in Materials and methods section. (B) Comparison of the estimated numbers of signature mutations in tumors with wild‐type vs. mutant *AHNAK*/*ERCC2*/*HRAS*. Data are plotted as mean ± SEM. *P* values were calculated using the Wilcoxon rank‐sum test. (C) Different mutation frequencies of the three genes among Asian/Black/White populations (140 Asian, 23 Black, and 324 White).

Consistent with previous reports [27], we showed that *ERCC2* mutation state and smoking were significantly correlated with ERCC2 & AGE signature activity (FDR < 0.1, *P* < 0.001, Fig. 2A,B). Interestingly, we also found that *ERCC2* mutation state was correlated with the activity of APOBEC‐b signature (FDR < 0.1), with an increase of 75 APOBEC‐b mutations in *ERCC2*‐mutant patients (Fig. 2B). ERCC2 is a key member of the nucleotide excision repair (NER) pathway and a previous study reported that mutations of ERCC2 resulted in loss of the cellular NER capacity [34]. It was possible that ERCC2 mutations may co‐operate with certain APOBEC enzymes to increase the mutagenicity of tumor cells during bladder cancer evolution. Notably, we identified that the APOBEC‐a signature activity, an indicator of good clinical outcome in bladder cancer [4], was associated with *AHNAK* mutations which appeared to regulate the TGFβ signaling in a previous study at the protein level [35] (Fig. 2A). The median numbers of APOBEC‐a mutations in patients with and without non‐silent *AHNAK* mutations were 235 and 62, respectively (Fig. 2B, *P* < 0.001). Increased APOBEC‐a signature activity may contribute to the accumulation of mutations in key regulator genes which may be positively selected during bladder cancer development. The inhibition or loss of TGFβ signaling was reported to shift cancer cells from error‐free homologous recombination DNA double‐strand break (DSB) to error‐prone alternative end‐joining (alt‐EJ) [36]. Correlation between *HRAS* mutation states and the MMR‐like signature activity was also noticed in our study (Fig. 2A,B). Recent report has noted that mutations in RAS/MAPK pathway genes exhibit strong selection and are enriched in replication repair‐deficient cancers [37]. However, detailed mechanism of this association is largely unknown. One possible mechanistic explanation is that mutations in RAS might be necessary for cancer cells to overcome the genomic instability.

We further validated that whether racial differences in mutation frequencies of several key genes or pathways may partially account for the racial differences in mutational signature activities among Asian/Black/White populations by combining datasets 1 and 2. Overall, the mutation frequencies of *AHNAK* and *HRAS* varied substantially among the different study populations. The frequency of *AHNAK* mutations was relatively high in the White patients who also showed the highest APOBEC‐a signature activity (9.6% in White vs. 4.3% in Black & 6.4% in Asian) while the frequency of *HRAS* mutations was the highest in the Asian patients (14.3% in Asian vs. 0.0% in Black & 3.4% in White) (Fig. 2C and Table S5). At the pathway level, genes involved in the DNA polymerase pathway and other conserved DNA damage response genes also had the highest mutation frequencies in White population (Fig. S5a).

To determine whether low‐frequency mutations in genes involved in the DNA damage responses were linked to mutational signature activities, we performed permutation tests for the DNA damage response pathways from curated databases (Materials and methods). We observed that mutations of the nucleotide excision repair (NER) pathway which includes the *ERCC2* gene were significantly associated with the activities of both ERCC2 & AGE and APOBEC‐b signatures (Fig. S5b). Mutations in the DNA polymerase pathway were identified to be associated with the APOBEC‐a signature activity. Additionally, mutations of a group of genes defined as other conserved DNA damage response genes were significantly associated with the mutagenesis of three signatures, APOBEC‐a, APOBEC‐b, and ERCC2 & AGE (Fig. S5b,c).

We next compared the expression levels of *AHNAK*, *ERCC2*, and *HRAS* among the three racial populations in Dataset 1. We observed that the expression levels of both *AHNAK* and *ERCC2* were significantly lower in Asian than those in White population (*P* < 0.05 and *P* < 0.001, respectively) (Fig. S6), which provided further evidence supporting that racial differences in the activities of *AHNAK* and *ERCC2* might contribute to the observed racial differences in the prevalence of APOBEC‐a and ERCC2 & AGE signatures in bladder cancer. However, *HRAS* expression showed no significant difference among different race groups (Fig. S6).

### 3.3 Racial disparities in cancer gene mutations and SCNAs

To depict the racial disparities in molecular features among different racial groups, we first identified the significantly mutated genes (SMGs) by combining all samples from the two study datasets and then compared the prevalence of mutations in SMGs among different race groups. A total of 57 SMGs were identified by MutSig2CV (*q* < 0.1) (Table S6) and 26 of them were cancer census genes that were expressed in over 75% of TCGA patients (supported by at least three read counts) and were kept for downstream analysis [38]. Five of the 26 SMGs had not been reported as SMGs in bladder cancer in previous studies [4, 6, 7, 8]: *CDKN1B* (1.2%), *ITK* (1.8%), *PRDM2* (3.1%), *FOXA1* (2.7%), and *BAP1* (1.8%). A total of 34 frequent arm‐level SCNAs occurred in at least 30% of patients by combining the two study datasets.

**Fig. 3:**
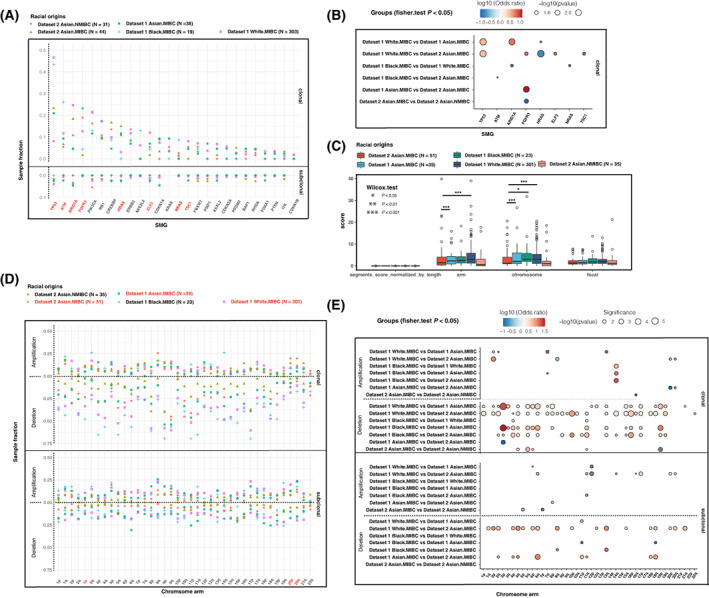
Racial difference in SMG (significantly mutated gene) mutations and SCNAs (somatic copy number alterations). (A) The prevalence of mutations in SMGs across races in dataset 1‐TCGA‐BLCA and dataset 2‐Chinese‐BLCA. (B) SMGs showing significantly different mutation frequencies in different populations. Only shown with Fisher test *P* value < 0.05. (C) The overall burden of SCNAs in different populations. *P* values were calculated using the Wilcoxon rank‐sum test: 0.01 < *P* < 0.05, *P* < 0.001 are shown as * and ***, respectively. Data are plotted as mean ± SEM. (D) The prevalence of SCNAs across race groups. Colored by racial origins. (E) Chromosome‐ or arm‐level SCNAs showing great racial disparities. Only shown with Fisher test *P* value < 0.05.

The prevalence of clonal mutations in serval SMGs varied substantially among different race groups. Both Black and White patients showed higher fractions of clonal *TP53* mutations than Asian patients from Datasets 1 and 2 (Odds ratio = 2.84–8.08, FDR < 0.05). The frequencies of clonal mutations in *HRAS* in Asian patients from Datasets 1 and 2 were consistently higher than Whites (Odds ratio = 0.16–0.26, FDR < 0.05). Compared with Asian patients, clonal mutations in *ATM* (Odds ratio = 4.69, FDR < 0.05) and *ARID1A* (Odds ratio = 3.06–5.90, FDR < 0.05) showed higher prevalence in Blacks and Whites, respectively. The frequency of *NRAS* mutations was higher in Blacks than Whites and three genes, including *FGFR3* (Odds ratio = 6.15, FDR < 0.05), *ELF3* (Odds ratio = 5.78, FDR < 0.05), and *TSC1* (Odds ratio, Not Applicable; FDR < 0.05), showed higher frequencies of clonal mutations in Whites than in Asian patients from Dataset 2. Interestingly, *FGFR3* clonal mutations were observed to occur more frequently not only in Asian patients in Dataset 1 than in Dataset 2. *FGFR3* mutations are the early driver events during NMIBC evolution and we also observed that *FGFR3* clonal mutations occurred more frequently in NMIBC than in MIBC in Dataset 2 patients [39] (Odds ratio = 0.09, FDR < 0.05) (Fig. 3A,B).

We next compared the overall burden of SCNAs at the chromosome/arm/focal levels across different racial groups (Materials and methods). NMIBC patients generally had a lower burden of SCNAs at the chromosome/arm levels than the MIBC patients. The overall arm‐ or chromosome‐level SCNA scores in Asian MIBC patients were lower than those in White or Black MIBC patients (Fig. 3C). This observation was consistent with the finding of low prevalence of clonal mutations in the *TP53* and *ATM* gatekeeper genes governing DNA repair in Asian MIBC patients. We further categorized each chromosome/arm‐level SCNA into clonal or subclonal event and found that the majority of arm‐level deletions either in clonal or subclonal states showed substantial inter‐population variations in their prevalence. A large number of chromosomes/arms showed higher prevalence of clonal deletions in White/Black MIBC patients than in the Asian MIBC patients. We also observed a large number of chromosomes/arms showing higher prevalence of subclonal SCNAs in Whites than in Asian patients from Dataset 2 (Fig. 3D). Moreover, we observed that only quite a few number of clonal arm‐level SCNAs, including del(3p) (Odds ratio = 0.08, FDR < 0.05) and amp(20) (Odds ratio = 0.11–0.22, FDR < 0.05), had higher prevalence, but a number of subclonal SCNAs showed significantly lower prevalence in Asian MIBC patients from Dataset 2 than in Asian patients from Dataset 1 (Fig. 3E).

It has been reported that bladder cancer patients with different genetic backgrounds may be presented with different disease stages at the time of diagnosis [10]. In our study datasets, we also observed that MIBC patients of Asian origins had higher prevalence of low‐stage disease while Blacks and Whites had higher prevalence of high‐stage disease (Fig. S7). To correct for the potential influence of heterogeneities in disease stages on the racial disparities in genomic features of MIBC, we further analyzed the racial differences in the mutation landscapes and SCNA profiles in low‐staged (staged I & II) MIBC patients due to the lack of sufficient numbers of high‐staged patients in the Asian datasets. Again, the prevalence of clonal mutations in *TP53* (Odds ratio = 3.14–4.25, FDR < 0.05) and *ATM* (Odds ratio = 8.77, FDR < 0.05) was significantly higher in Whites and Blacks than in Asians, respectively, among MIBC patients with low‐stage disease (Fig. S8). The prevalence of clonal mutations in *NFE2L2* (Odds ratio = 55.45, FDR < 0.05) was the highest in low‐staged Black patients and the fractions of White patients harboring clonal mutations in *FGFR3* (Odds ratio = 10.31, FDR < 0.05), *TSC1* (Odds ratio, Not Applicable; FDR < 0.05), and *CDKN2A* (Odds ratio, Not Applicable; FDR < 0.05) were significantly higher than those of Asian patients from Dataset 2. Clonal mutations in *HRAS* also showed significantly higher prevalence in Asians than in Whites among the low‐staged patients. We also confirmed that the prevalence of *FGFR3* (Odds ratio = 14.17, FDR < 0.05) clonal mutations was significantly higher in Asians from Dataset 1 than in Asians from Dataset 2 among the low‐stage MIBC patients. The trends of higher prevalence of clonal deletions and subclonal SCNAs in Whites or Blacks were also observed among patients with low‐stage MIBC. Differences in the frequencies of clonal deletions of several chromosome arms including del(3p) and subclonal SCNAs affecting a number of other arms were also observed between Asians from Datasets 1 and 2 among the low‐staged patients.

### 3.4 Genomic architectures and subtypes of bladder cancer

**Fig. 4:**
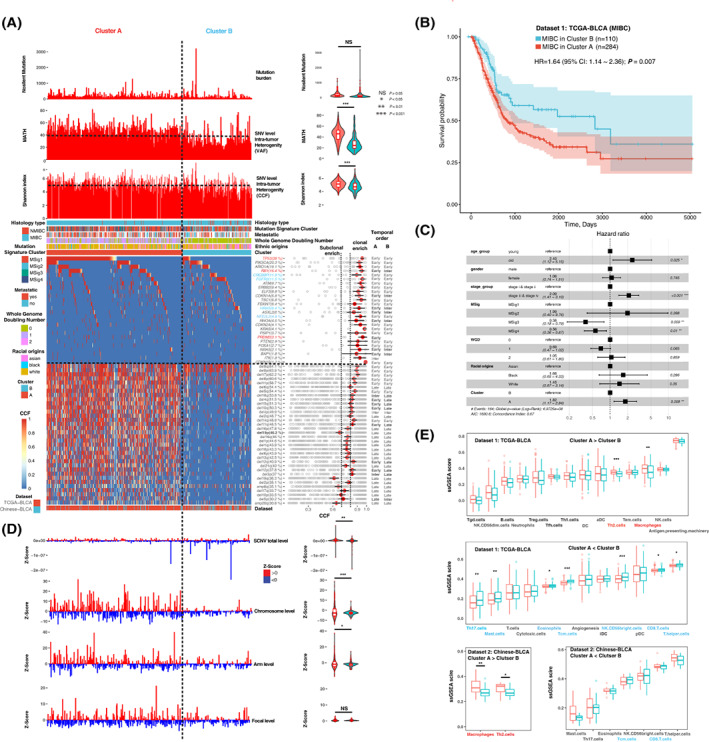
Prognostic subtypes of bladder cancer with distinct genomic architectures. (A) Landscape of genomic architectures in 505 bladder cancer samples. Cluster A and Cluster B of bladder cancer patients were subtyped by the NMF (non‐negative matrix factorization) ‘lee’ algorithm based on the CCFs of 26 SMGs and frequent arm‐level SCNAs across 505 bladder cancer samples. Data are plotted as mean ± SEM. (B) Kaplan–Meier survival analysis of the two clonal subtypes. Only using TCGA dataset due to the lack of some key clinical information in Chinese dataset (C) multivariate Cox regressions analysis of survival by including clonal subtype, racial origins, WGD (whole genome doubling), MSig cluster, age, gender, and TNM stage. Data are plotted as mean ± SEM. (D) Differences in the genome‐wide/chromosome/arm and focal levels of SCNAs scores between two subtypes. The calculation of the SCNA scores in different levels is followed by a previous study with minor modifications. Details in *calculation of the SCNA scores* in Materials and methods section. Data are plotted as mean ± SEM. (E) Immune features of the two subtypes (red: Cluster a, blue: Cluster B). The infiltration levels of different types of immune cells could be inferred by the ssGSEA score, using published cell type‐specific genes of immune cells. *P* values were calculated using the Wilcoxon rank‐sum test: 0.01 < *P* < 0.05, 0.001 < *P* < 0.01, *P* < 0.001 are shown as *, ** and ***, respectively. Data are plotted as mean ± SEM.

Our study included a large number of bladder cancer patients with different genetic backgrounds and we then tried to perform molecular subtyping of bladder cancer by integrating multiple somatic events according to their CCFs in all samples by combining Datasets 1 and 2. We inferred the genomic architectures of bladder cancer by focusing on the 26 SMGs with reported roles in the Cancer Gene Census database and the 34 frequent arm‐level SCNAs altered in at least 30% of patients as defined above. To explore the contribution of these SMGs and frequent arm‐level SCNAs during bladder cancer evolution, we estimated the CCF of each SMG and SCNA in each sample. Overall, the median CCFs of SMGs were higher than those of SCNAs by combining the two study datasets (Fig. 4A). All the 26 SMGs were found to be enriched with clonal non‐silent mutations (FDR < 0.1) while only about 50% (17/34) and 15% (5/34) of the frequent arm‐level SCNAs were significantly enriched with clonal and subclonal events (FDR < 0.1), respectively (Fig. 4A). These data suggested that heterogeneities in the genomic architectures of bladder cancer were more likely to arise from diversity in the clonality of arm‐level SCNAs.

Few previous studies evaluated the prognostic values of individual somatic events according to their clonal or subclonal states in bladder cancer. Nevertheless, somatic alterations affecting each individual SMG and SCNA usually could only be detected in a minor fraction of samples and stratification of them into clonal or subclonal events would further reduce the numbers of patients harboring alterations in prognosis analysis. We did not observe significant differences in the clinical outcomes of different racial groups in Dataset 1, which may be partially due to the lack of sufficient number of Asian and Black patients with their survival information available (Fig. S9). Thus, we further evaluated the prognostic value of the SMGs and frequent SCNA events according to their clonal or subclonal states by ignoring the racial origins of the patients to increase the sample sizes. We combined the different racial groups in Dataset 1 together in Kaplan–Meier analysis and multivariate Cox regression analysis. In total, we identified only four individual events with potential prognostic value in Dataset 1 (Table 1, Table S7). Dataset 1 patients with *KRAS* mutations, all of which were clonal, had poor clinical outcome while clonal deletions of either 21q or 12p and subclonal deletions of 18p were associated with shortened overall survival in Dataset 1.

**Table 1:** Distinct prognostic values of the clonal and subclonal events.

Variable	Mut vs. wild	Clonal Mut vs. wild	Subclonal Mut vs. wild
*KRAS*	*N* = 15	*N* = 15	NA
Univariate analysis	HR = 2.31 (1.22–4.38) ***P* = 0.01**	HR = 2.31 (1.22–4.38) ***P* = 0.01**	
Multivariate analysis:age, gender, TNM stage	HR = 2.91 (1.52–5.59) ***P* = 0.001**	HR = 2.91 (1.52–5.59) ***P* = 0.001**	NA
*Del18p*	*N* = 182	*N* = 119	*N* = 63
Univariate analysis	HR = 1.50 (1.1–2.05) ***P* = 0.01**	HR = 1.34 (0.949–1.89) *P* = 0.10	HR = 1.91 (1.27–2.86) ***P* = 0.002**
Multivariate analysis:age, gender, TNM stage	HR = 1.35 (0.99–1.85) *P* = 0.06	HR = 1.21 (0.856–1.72) *P* = 0.28	HR = 1.7 (1.12–2.58) *P* = 0.01
*Del21q*	*N* = 167	*N* = 103	*N* = 64
Univariate analysis	HR = 1.42 (1.04–1.92) ***P* = 0.03**	HR = 1.45 (1.03–2.05) ***P* = 0.03**	HR = 1.36 (0.896–2.05) *P* = 0.15
Multivariate analysis:age, gender, TNM stage	HR = 1.29 (0.949–1.76) *P* = 0.10	HR = 1.35 (0.954–1.91) *P* = 0.09	HR = 1.2 (0.788–1.82) *P* = 0.40
*Del12p*	*N* = 156	*N* = 114	*N* = 42
Univariate analysis	HR = 1.35 (0.993–1.83) *P* = 0.06	HR = 1.55 (1.12–2.14) ***P* = 0.009**	HR = 0.881 (0.516–1.5) *P* = 0.64
Multivariate analysis:age, gender, TNM stage	HR = 1.19 (0.877–1.63) *P* = 0.26	HR = 1.35 (0.972–1.89) *P* = 0.07	HR = 0.824 (0.481–1.41) *P* = 0.48

*Note*: Bold indicates *P* value < 0.05.

We next tried to stratify whether there were any subtypes of bladder cancer whose overall genetic architectures had great influence on their clinical outcomes. We performed NMF analysis of the CCFs of the 26 SMGs and 34 arm‐level SCNAs by combining the two study datasets. In total, we identified two molecular subgroups (termed as clusters A and B), whose clinical outcomes were divergent. Because long‐term survival information was not available for the patients from Dataset 2, we performed survival analysis for all the MIBC samples in the TCGA dataset. TCGA MIBC patients in cluster A had worse survival rate than those in cluster B (Fig. 4B; HR = 1.64, 95% CI: 1.14–2.36; *P* = 0.007). Multivariate analysis further proved that the genomic architectures could predict the clinical outcomes of bladder cancer patients independently by adjusting for age, gender, TNM stage, and mutational signature (Fig. 4C). Further comparison of these two genomic subtypes with the molecular subtypes defined by TCGA at different expression levels showed that genomic cluster B tended to be clustered with the molecular subtypes mRNA_Luminal_papillary, lncRNA_3, miRNA_3, and RPPA_1 in Dataset 1 patients [4] (Fig. S10). It had been shown previously that bladder cancer patients exhibiting the Luminal_papillary signature at the mRNA level had low risk of disease progression [4]. Patients belonging to either the miRNA subtype 3 which was correlated with the lncRNA_3 signature or the RPPA cluster 1 showed the best survival in previous TCGA analysis [4]. Overall, our analysis at the genomic level provided further evidence complementary to the previously defined molecular subtypes of bladder cancer at the expression levels.

### 3.5 Molecular features of prognostic subtypes defined by genomic architectures

To understand which factors contributed to the distinct prognosis of bladder cancer subtypes defined by genomic architectures, we compared the prevalence of 60 potential driver events (26 SMGs and 34 arm‐level SCNAs) between clusters A and B. We showed that four SMGs (*CREBBP*, *FGFR3*, *HRAS*, and *NFE2L2*) but none of the frequent arm‐level SCNAs had significantly higher prevalence of genetic aberrations in cluster B patients who had better prognosis than in cluster A patients (FDR < 0.1, Fig. 4A and Table S8). Three SMGs (*TP53*, *RB1*, and *PRDM2*) and about 91% of the frequent arm‐level SCNAs were enriched with alterations in cluster A patients who showed poor prognosis (FDR < 0.1, Fig. 4A and Table S8). Notably, of the SMGs mutated more frequently in cluster B than in cluster A, three showed race‐specific enrichment of clonal mutations among the low‐staged patients with *FGFR3*, *HRAS*, and *NFE2L2* mutations being enriched in Whites/Asians from Dataset 2, Asians from the two study datasets and Blacks, respectively (Fig. 4A). These observations indicated that trans‐ancestry analysis of the genomic architecture of bladder cancer may be effective in screening out the trans‐ancestry prognostic subtypes.

We inferred the potential temporal ordering of the acquirements of SMGs and frequent arm‐level SCNAs in clusters A and B patients during bladder cancer evolution (Materials and methods). Among cluster A patients, 25 of 26 SMGs and 21 of 34 arm‐level SCNAs were acquired at the early and intermediate/late evolution stages, respectively (Fig. 4A). Among cluster B patients, 17 of 24 SMGs and 27 of 34 arm‐level SCNAs were acquired at early and intermediate/late evolution stages, respectively (Fig. 4A). These data further supported that the clonal diversities of both clusters A and B patients were largely contributed by the large‐scale SCNAs acquired at late evolution stages. Several SCNAs, including del(9), del(17p), del(8p), and del(11p), were early clonal events shared by patients in both clusters A and B while arm‐level SCNAs, including del(4q), del(15q), del(10q), del(11q), and del(12), were acquired at the early and late stages in clusters A and B, respectively (Fig. 4A).

To explore whether there were any other differences in the overall genomic landscapes between clusters A and B patients, we firstly compared their non‐silent somatic mutation burdens and found no significance difference. The extent of intra‐tumor heterogeneities as measured by MATH values and Shannon entropy of CCFs in cluster A patients was significantly higher than that of cluster B patients (*P* < 0.001, Fig. 4A and Fig. S11). We next estimated the overall burden of SCNAs at the chromosome/arm/focal levels for each patient and showed that cluster A patients had significant higher burden of large‐scale SCNAs (arm & chromosome levels) but not focal SCNAs (*P* < 0.001, Fig. 4D). Whole‐genome doubling (WGD) events were prevalent in cluster A and patients with Asian origin were enriched in cluster B (both *P* < 0.001). To be more specific, although our datasets included only a small number of NMIBC patients from Dataset 2, cluster B was enriched with Dataset 2 NMIBC patients (51.0% in cluster B vs. 22.9% in cluster A, *P* < 0.005) and Dataset 1 MIBC patients who were less likely to develop metastatic disease (52.6% in cluster B vs. 33.3% in cluster A, *P* < 0.001, Fig. 4A). Further analysis of the overall survival for Dataset 1 MIBC patients showed that patients in cluster A had worse survival rate than those in cluster B after adjusting for WGD, racial origins as well as other clinical and genomic parameters (Fig. 4C). These observations suggested that it was feasible to distinguish the heterogeneous MIBC patients into two subtypes with distinct clinical outcomes based on their inherited genomic architectures.

To further study whether high intra‐tumor heterogeneity and chromosomal instability of cluster A patients had influence on the tumor microenvironment, we compared the relative expression levels of the immune signature genes among two genomic prognostic clusters using ssGSEA (Fig. 4E). In Dataset 1, the infiltrating levels of Th17 cells and CD8^+^ T cells were observed to be significantly higher in cluster B patients (*P* < 0.001 and FDR < 0.1, Fig. 4E). Previous studies proved that Th17 cells as subsets of T helper lymphocytes can mediate the antitumor immune responses through interacting with effector CD8^+^ T cells [40]. Cluster A patients had significantly higher levels of infiltrating Th2 cells and macrophages (Both *P* < 0.001 and FDR < 0.1, Fig. 4E), which had been proved to facilitate tumor growth and metastasis leading to the reduced survival in the cancer [41, 42, 43]. These observations on differences in tumor microenvironment between clusters A and B were also validated independently in the Chinese dataset (Fig. 4E).

## 4 Discussion

Bladder cancer, a heterogeneous group of diseases with high morbidity and mortality all over the world, still lacks effective treatments and prognostic indicators. Copious epidemiological evidence demonstrates the existence of great racial disparities in the incidence and prognosis of bladder cancer. However, there were few studies investigating the molecular features of bladder cancer patients across different racial populations. Mutational signatures are reflective of previous exposures to various exogenous and endogenous mutagenic processes during the lifetime of cancer evolution. The observed enrichment of clonal mutations in APOBEC‐a signature in our study further confirmed the tumorigenic roles of this signature during the early stages of bladder cancer development [5, 44, 45].

Notably, we also observed that the activities of several mutational signatures varied substantially among different racial populations. Racial differences in exposures to exogenous factors related to life history may account for some of the observed racial differences in mutational signatures. The ARISTOLOCHIC signature could only be detected in some Asian patients, especially those from Dataset 2. Herbs or products containing aristolochic acids are widely consumed in some areas in Asia and the ARISTOLOCHIC signature had been detected in several human cancers, including bladder cancer, renal cell carcinoma, and liver cancer, in previous studies in Asian patients [46, 47, 48]. Our observation that the ARISTOLOCHIC signature showed significant enrichment of subclonal mutations indicated that this life history‐related signature was less likely to play important roles during the early development of bladder cancer. In terms of the newly identified MMR‐like signature which also significantly enriched with subclonal mutations, more evidence was needed to further exclude the potential influence of study batch effects on the observation of this signature in a single study dataset.

Interestingly, our study also suggested the possible roles of racial differences in the prevalence of gene mutations in mediating the racial disparities in the activities of mutational signatures. It had been shown that mutations in *ERCC2* were associated with the activities of COSMIC signature 5 in bladder cancer previously [27]. We showed that mutations in *ERCC2* and other SMGs or pathways were associated with the activities of mutational signatures in bladder cancer. APOBEC‐a signature showed varied activities among different patient groups and we provided preliminary evidence suggesting that the high APOBEC‐a signature activity was likely associated with the high prevalence of somatic mutations in *AHNAK* in patients from the White population. Future studies are needed to characterize the biology function of the associations between mutations in SMGs or pathways and mutational signature activities to establish the potential of utilizing the mutated genes such as *AHNAK* as biomarkers for the clinical management of bladder cancer patients.

Previous studies had shown that high APOBEC‐a signature activity was indicative of good prognosis [4, 7, 8]. In our analysis, we showed that clustering of the patients based on their mutational signatures could stratify the patients into different prognostic subgroups. Batch effects may have some influence on interpreting the results of cluster analysis of mutational signatures in patients from different study sources. However, prognostic analysis was performed for patients from Dataset 1 only in our study, which means that all the results of survival analysis were less likely to be affected by batch effects. Although survival information was not available for the NMIBC patients from Dataset 2, it had been well known that NMIBC patients usually can survive with the disease for quite a long time. We assumed that the observed clustering of a subset of Dataset 1 MIBC patients who had better survival with NMIBC patients from Dataset 2 in prognostic analysis cannot be explained by batch effects solely.

Trans‐ancestry analysis of the genomic features of bladder cancer revealed that all the SMGs were enriched with clonal mutations, but a large number of arm‐ or chromosome‐level SCNAs were subclonal events. Thus, clonal defects of the SMGs may play pivotal roles in the early evolution stages of bladder cancer. Clonal mutations in SMGs (such as *TP53* and *ATM*) regulating the DNA repair machinery further lead to the accumulation of an abundant of SCNAs which provides the rich resources fostering the clonal diversity and inter‐population heterogeneity of bladder cancer. The prevalence of *TP53* and *ATM* clonal mutations varied greatly among Whites/Blacks/Asians, which may contribute to the diversified overall burden of SCNAs as well as the inter‐populational differences in the prevalence of individual clonal/subclonal SCNA events. Interestingly, when we performed trans‐ancestry molecular subtyping based on the genomic architectures of patients from different populations, we identified a trans‐ancestry prognostic subtype that was enriched with Asian NMIBC patients and White/Black/Asian MIBC patients who tend to harbor defects in driver genes, such as *FGFR3*, *HRAS*, and *NFE2L2*, showing significant racial differences in mutation prevalence among low‐staged patients. These findings demonstrated the feasibility of identifying trans‐ancestry prognostic or even therapeutic subtypes based on their genomic architectures, despite of the fact that the genomic features of bladder cancer varied greatly among different populations.

The long‐term survival rates for the MIBC patients are generally much lower than the NMIBC patients who usually suffer from disease recurrence but rarely die of bladder cancer [39]. To reduce the risk of developing metastatic disease, MIBC patients are routinely treated with cystectomy of the bladder, which is much more radical and invasive than transurethral resection of bladder tumor currently used for treating NMIBC. Thus, it is imperative to identify molecular markers or subtypes to predict the prognosis of MIBC for guiding the clinical choice of treatments to improve the life quality of bladder cancer patients. Our study datasets were mainly composed of MIBC patients from two studies’ datasets with different racial backgrounds and a small number of NMIBC patients from a single dataset were also used as the external control for stratifying the MIBC patients into prognostic subtypes. Only a few clonal or subclonal somatic events, including SMGs and arm‐level SCNAs, were potentially associated with the prognosis of MIBC in our study. Previous studies showed evidence suggesting that a subset of MIBC patients shared similar molecular features with the NMIBC patients whose genetic landscapes were characterized by high prevalence of mutations in the FGFR3‐Ras pathway and low burden of SCNAs [49]. Molecular subtypes of MIBC exhibiting similarities to NMIBC at the mRNA expression level had been reported previously, but the genetic causes underlying the dynamic changes at expression levels between subtypes are not clear [4]. We analyzed the genomic architectures of MIBC and NMIBC patients and selected the clonal/subclonal states of 60 potential driver events as features of the prognostic subtypes. We identified two prognostic clusters of MIBC, with the shorter overall survival subtype showing higher burden of large‐scale SCNAs and WGD, higher prevalence of mutations in *TP53* and *RB1*, higher levels of intra‐tumor heterogeneity as well as suppressed tumor microenvironment and higher rate of developing metastatic disease. These findings highlighted the possibility of directing the clinical management of MIBC patients based on their genomic architectures.

There are several limitations in this study. Although we applied a unified pipeline throughout the analysis, including processing raw data and genomic variant calling, batch effects caused by the different designs of the two datasets may not be fully eliminated. While the populations differ in genetic ancestry, they also vary substantially in diet and environment. We had not taken these factors into full consideration in this study. Additionally, self‐reported ancestry in TCGA Dataset is used. Self‐reported Asian samples in TCGA are likely a heterogeneous mix of Asian ancestries, which may not directly comparable to the Chinese Asian sample. Taken together, we concluded that there were great racial disparities in the genomic architecture of bladder cancer and the confounding factors would not affect the main conclusions of our study. The identification of a trans‐ancestry prognostic subtype with distinct genomic features and good clinical outcome, which could be validated in different datasets and disease subtypes, may be useful for guiding personalized treatment for bladder cancer and provides an excellent resource for other studies.

## 5 Conclusion

In summary, our study identifies the heterogeneity of genomic features of bladder cancer patients across different racial populations and suggests their potential causes in great racial disparities in the incidence and prognosis of bladder cancer. We also identify a trans‐ancestry prognostic subtype with distinct genomic features and good clinical outcome. These findings may be useful for guiding personalized treatment for bladder cancer.

## Conflict of interest

The authors declare no conflict of interest.

## Author contributions

BFZ, X‐yG, and YH conceived the study. BFZ and XCL performed the bioinformatics analysis. GSP, PLJ, JYW, XY, and ZMZ downloaded and processed TCGA‐BLCA data. CXW and SMP performed GSEA analysis. BFZ and YH wrote the manuscript. BFZ, JYW, XY, X‐yG, and YH revised the manuscript.

### Peer review

The peer review history for this article is available at https://publons.com/publon/10.1002/1878‐0261.13360.

## Supporting information


**Fig. S1.** Mutation signatures identified by the Bayesian NMF algorithm.
**Fig. S2.** The distribution of activities and fractions of the five mutation signatures in bladder cancer patients.
**Fig. S3.** Comparison of the activities of the five mutation signatures in Asian bladder cancer patients from Dataset 1 and Dataset 2.
**Fig. S4.** The decomposition of mutation signatures in bladder cancer patients with the non‐negative least square method.
**Fig. S5.** Defects in DNA repair pathways associated with signature activities.
**Fig. S6.** The expression levels of *ERCC2/AHNAK/HRAS* genes in Asian/Black/White bladder cancer patients.
**Fig. S7.** The distribution of age/TNM stage/gender in bladder cancer patients with different ethnicities.
**Fig. S8.** Ethnic differences in SMG mutations and SCNAs in patients with Stage I & II disease.
**Fig. S9.** Survival analysis of Asia/Black/White bladder cancer patients.
**Fig. S10.** Comparison of the two genomic subtypes (Clusters A and B) defined in our study with the molecular subtypes defined by TCGA at different expression levels.
**Fig. S11.** The correlation analysis of MATH score with tumor purity, arm/chromosome‐level SCNA score.

**Table S1.** Clinical characteristics of patients used for this study.

**Table S2.** Clinical characteristics associated with the two subtypes of bladder cancer.

**Table S3.** Statistics of somatic single nucleotide variants (SNVs), insertions/deletions (Indels), and somatic copy number alterations (SCNAs).

**Table S4.** Comparison of mutational signatures identified in our study to COSMIC mutational signatures (related to Fig. 1A).

**Table S5.** Mutation table of the *AHNAK/ERCC2/HRAS* genes among Asian/Black/White populations (Related to Fig. 2c)

**Table S6.** Significantly mutated genes (SMGs) identified by MutSig2CV.

**Table S7.** Kaplan–Meier survival analysis result of all SMGs and frequent SCNAs events according to their clonal or subclonal versus wild state.

**Table S8.** Predominance of mutation acquisitions in patients with cluster A and B bladder cancer patients.

## Data Availability

Any relevant data are available from the authors upon reasonable request. The raw sequencing data used in this manuscript are all publicly available (SRA063495 and Genomic Data Commons (GDC) data portal (http://gdc‐portal.nci.nih.gov)). The data produced by the analysis in this manuscript are summarized in the supplementary tables. All custom code used in this work is available from the corresponding authors upon reasonable request.
