# Supplementary material for: Integrated analysis of racial disparities in genomic architecture identifies a trans‐ancestry prognostic subtype in bladder cancer
Source: Mol Oncol. 2022 Dec 29;17(4):564–81. doi: 10.1002/1878-0261.13360 (PMC10061287; doi:10.1002/1878-0261.13360)
Supplement: Supplementary file 1 — Fig. S1. Mutation signatures identified by the Bayesian NMF algorithm. Fig. S2. The distribution of activities and fractions of the five mutation signatures in bladder cancer patients. Fig. S3. Comparison of the activities of the five mutation signatures in Asian bladder cancer patients from Dataset 1 and Dataset 2. Fig. S4. The decomposition of mutation signatures in bladder cancer patients with the non‐negative least square method. Fig. S5. Defects in DNA repair pathways associated with signature activities. Fig. S6. The expression levels of ERCC2/AHNAK/HRAS genes in Asian/Black/White bladder cancer patients. Fig. S7. The distribution of age/TNM stage/gender in bladder cancer patients with different ethnicities. Fig. S8. Ethnic differences in SMG mutations and SCNAs in patients with Stage I & II disease. Fig. S9. Survival analysis of Asia/Black/White bladder cancer patients. Fig. S10. Comparison of the two genomic subtypes (Clusters A and B) defined in our study with the molecular subtypes defined by TCGA at different expression levels. Fig. S11. The correlation analysis of MATH score with tumor purity, arm/chromosome‐level SCNA score. [file MOL2-17-564-s001.pdf]

Racial differences in genomic features and new trans-ancestry prognosis subtypes in bladder cancer

Zhang et al.

Supplementary Fig 1

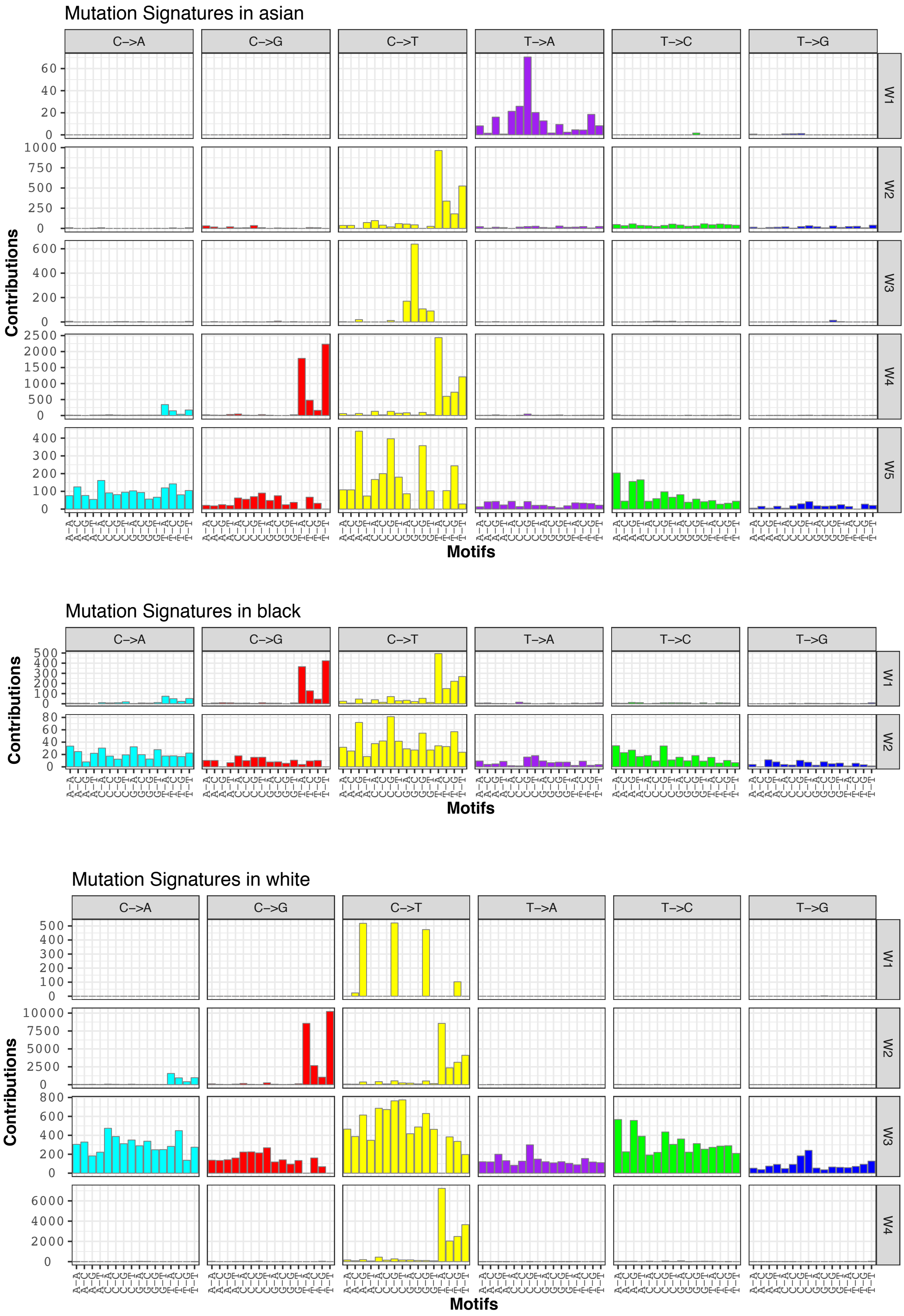

Supplementary Figure 1. Mutation signatures identified by the Bayesian NMF algorithm. From top to bottom: Asian, Black and White. X-axis: 96 mutation type. Y-axis: contributions or activity of each mutation type.

Supplementary Fig 2

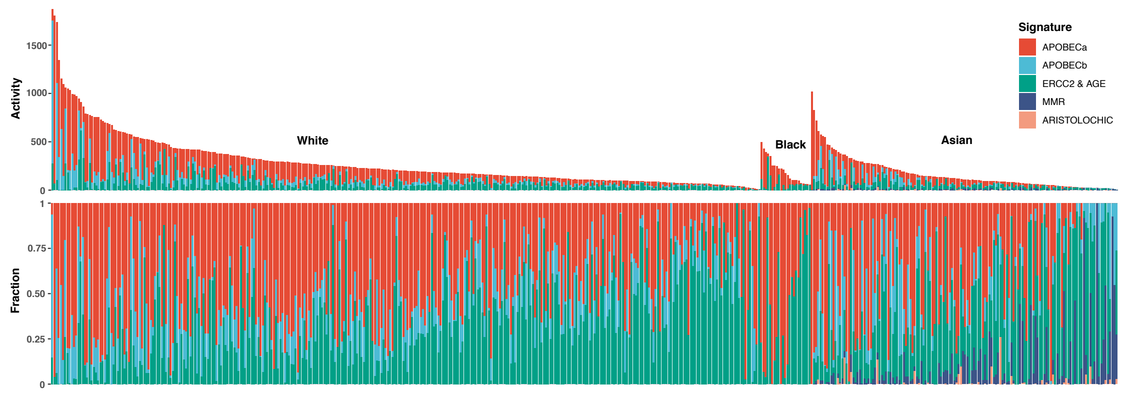

Supplementary Figure 2. The distribution of activities and fractions of the five mutation signatures in bladder cancer patients. Each color represents each mutational signature.

Supplementary Fig 3

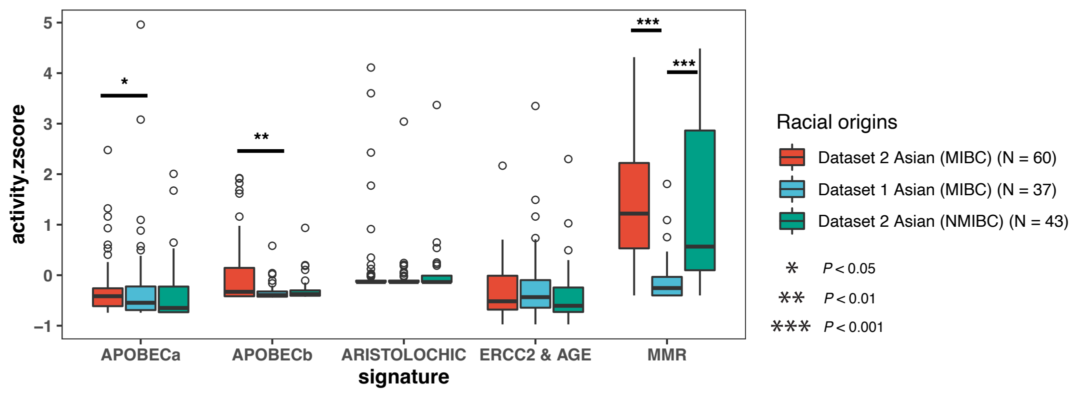

Supplementary Figure 3. Comparison of the activities of the five mutation signatures in Asian bladder cancer patients from Dataset 1 and Dataset 2. X-axis: mutation signature. Y-axis: scaled activity score of mutational signatures in each racial origin group. *P* values were calculated using the Wilcoxon rank-sum test: $0.01 < P < 0.05$, $0.001 < P < 0.01$, $P < 0.001$ are shown as *, ** and ***, respectively. Data are plotted as mean ± SEM.

Supplementary Fig 4

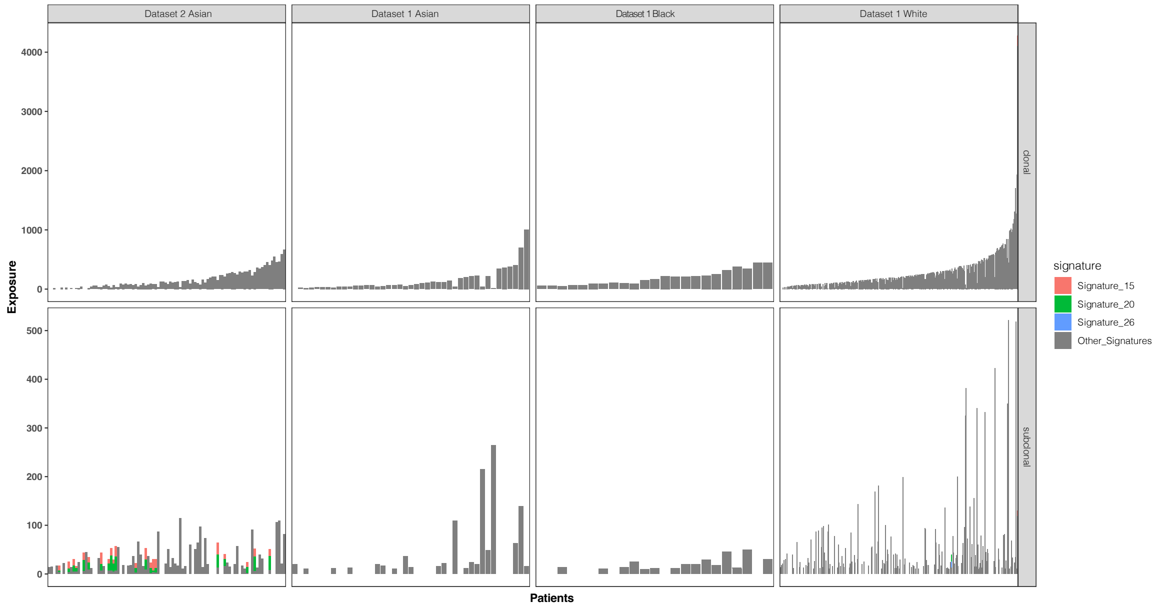

Supplementary Figure 4. The decomposition of mutation signatures in bladder cancer patients with the non-negative least square method. X-axis: each bar represents one patient. Y-axis: exposure of mutational signature in each patient.

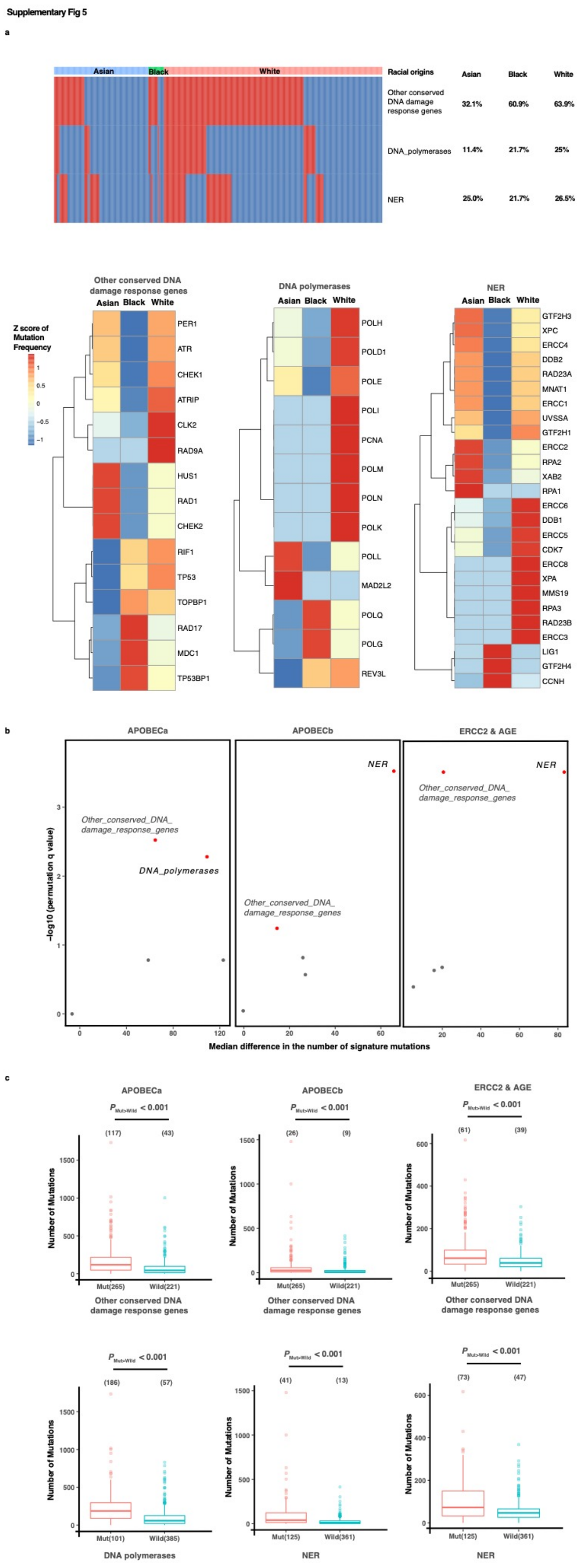

Supplementary Figure 5. Defects in DNA repair pathways associated with signature activities. (a) Differences in the mutation frequencies of the three pathways among Asian/Black/White populations. (b) Mutation enrichment analysis identifies associations between three pathways and signature activities. (c) Comparison of the estimated numbers of signature mutations in tumors with wild-type versus mutant genes in the three pathways.

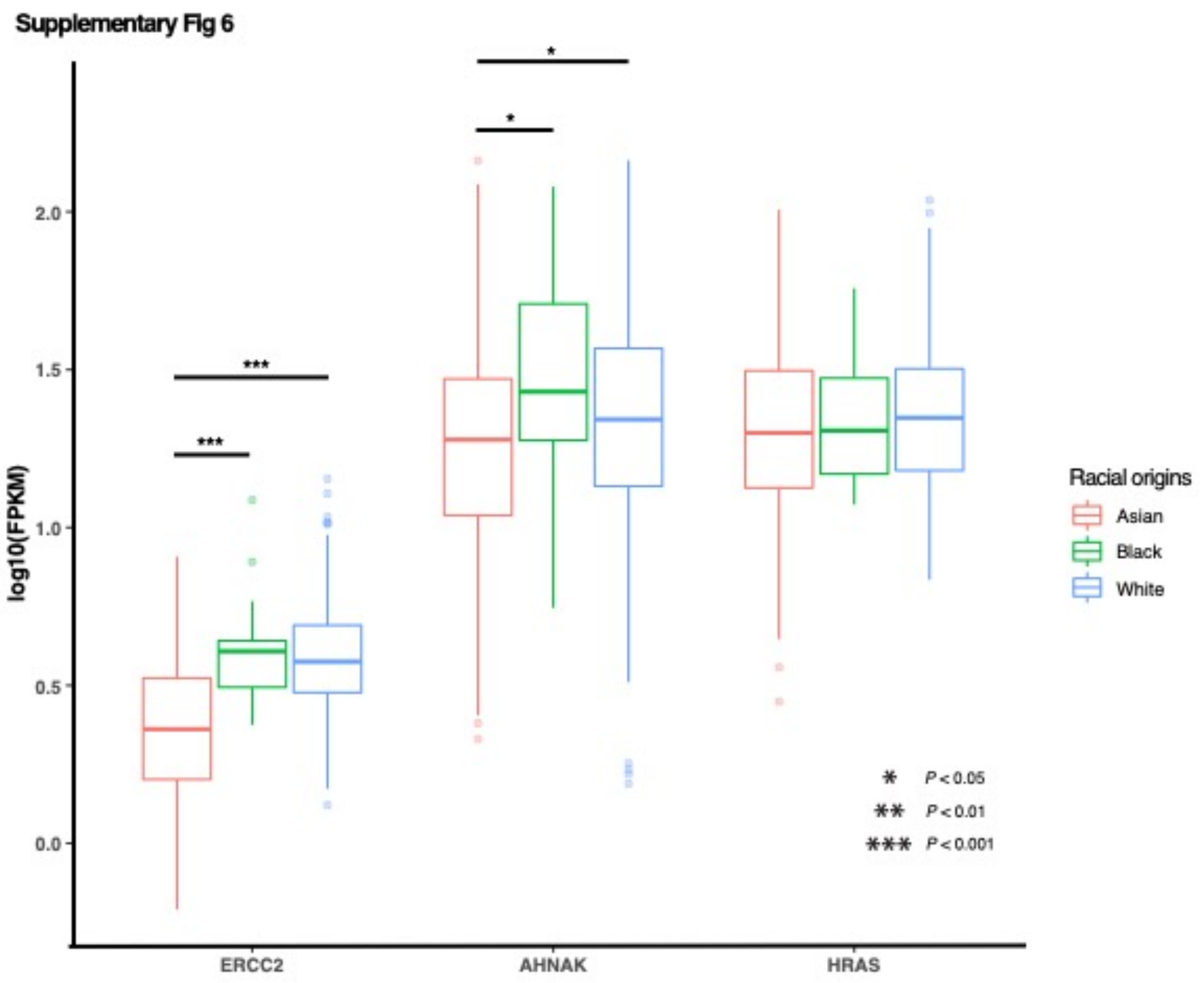

Supplementary Figure 6. The expression levels of *ERCC2/AHNAK/HRAS* genes in Asian/Black/White bladder cancer patients. *P* values were calculated using the Wilcoxon rank-sum test: 0.01 < P < 0.05, 0.001 < P < 0.01, P < 0.001 are shown as *, ** and ***, respectively. Data are plotted as mean ± SEM.

Supplementary Fig 7

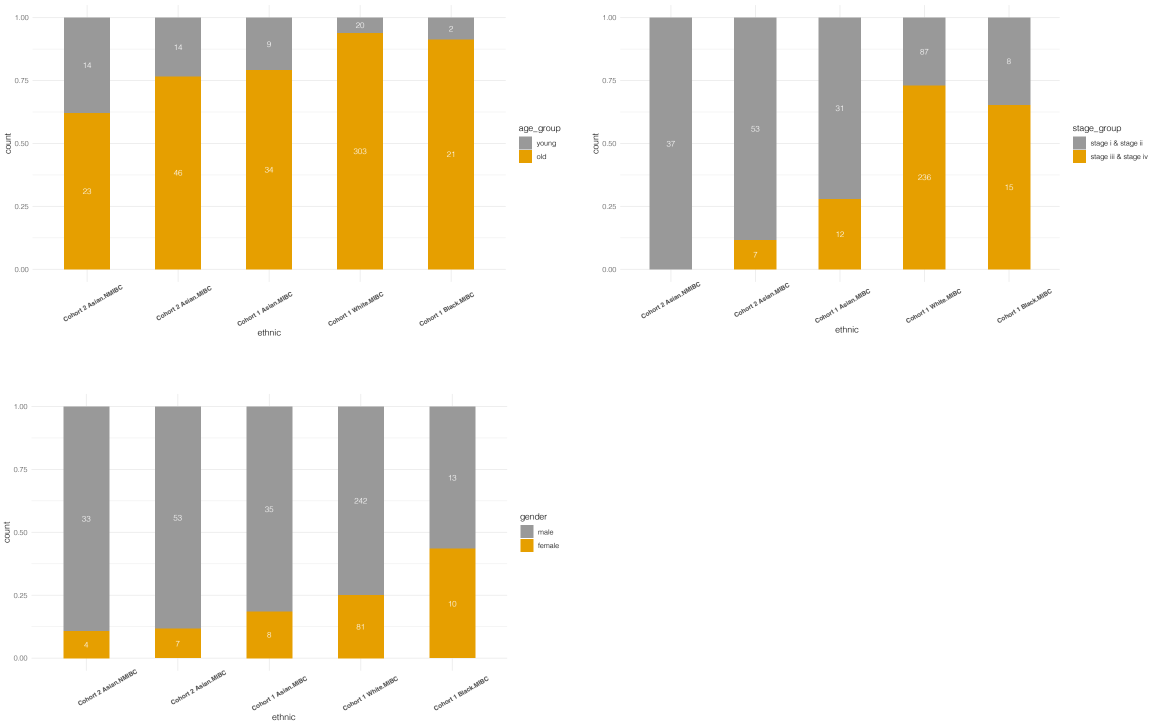

Supplementary Figure 7. The distribution of age/TNM stage/gender in bladder cancer patients with different ethnicities. Cohort 1: TCGA-BLCA. Cohort 2: Chinese-BLCA.

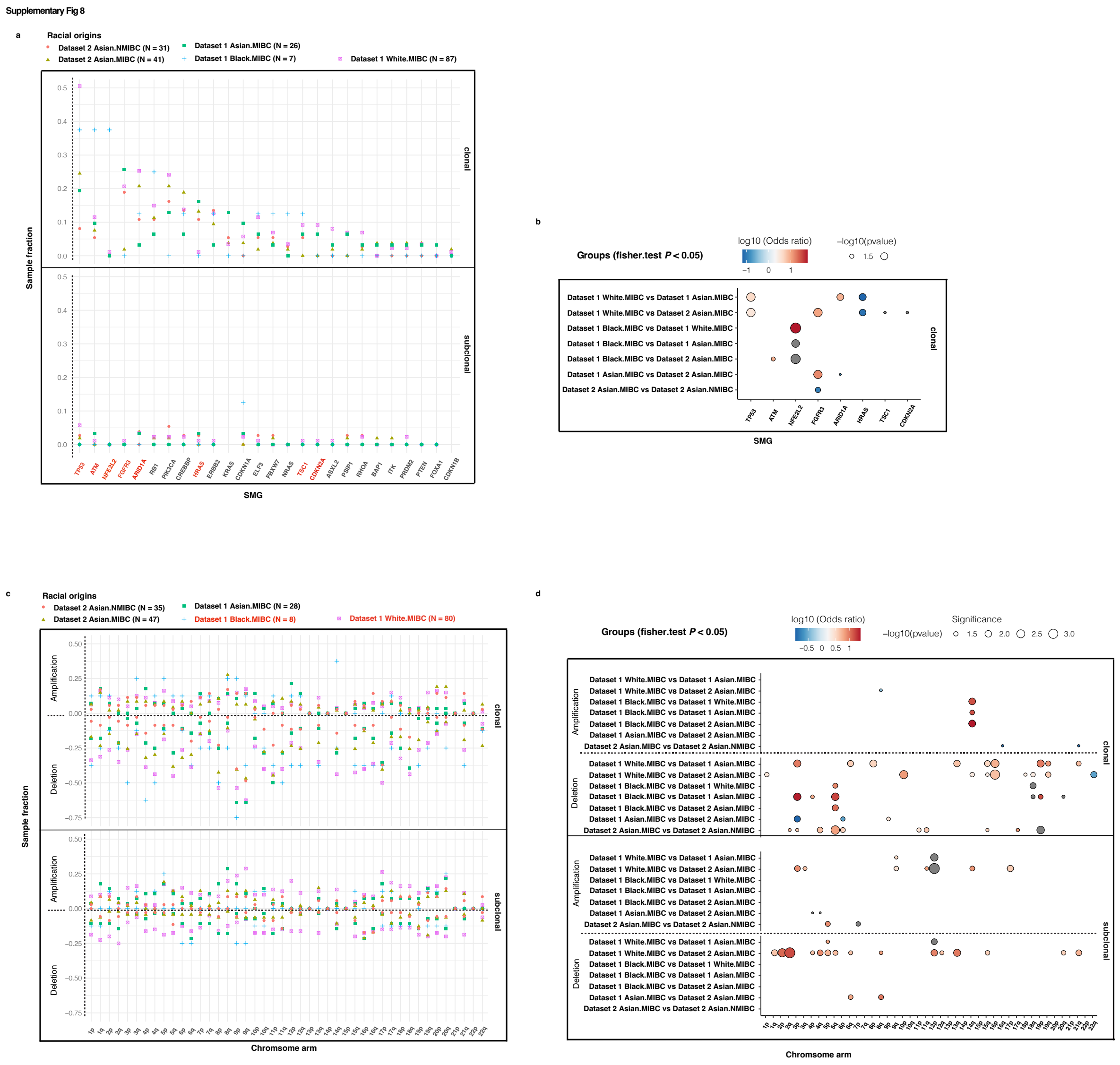

Supplementary Figure 8. Ethnic differences in SMG mutations and SCNAs in patients with Stage I & II disease. (a) The prevalence of mutations in SMGs across racial origins. (b) SMGs showing significantly different mutation frequencies in different racial origins. (c) The prevalence of SCNAs across racial origins. (d) Chromosome- or arm-level SCNAs showing great racial disparities.

Supplementary Fig 9

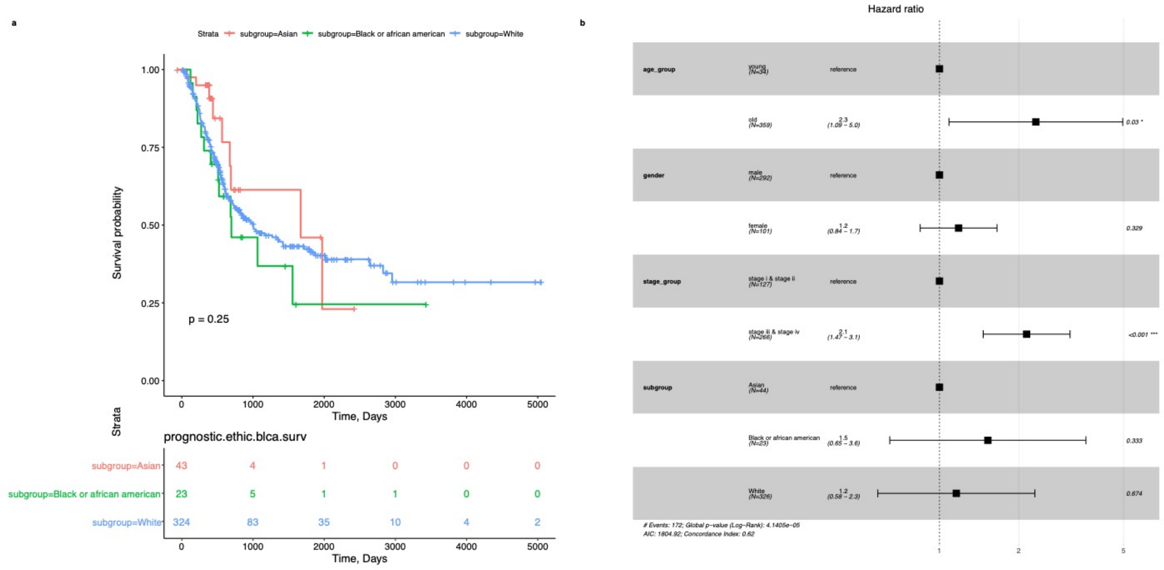

Supplementary Figure 9. Survival analysis of Asia/Black/White bladder cancer patients. (a) Kaplan-Meier survival analysis. (b) Multivariate Cox regressions analysis of survival by including racial origins, age, gender and TNM stage.

Supplementary Fig 10

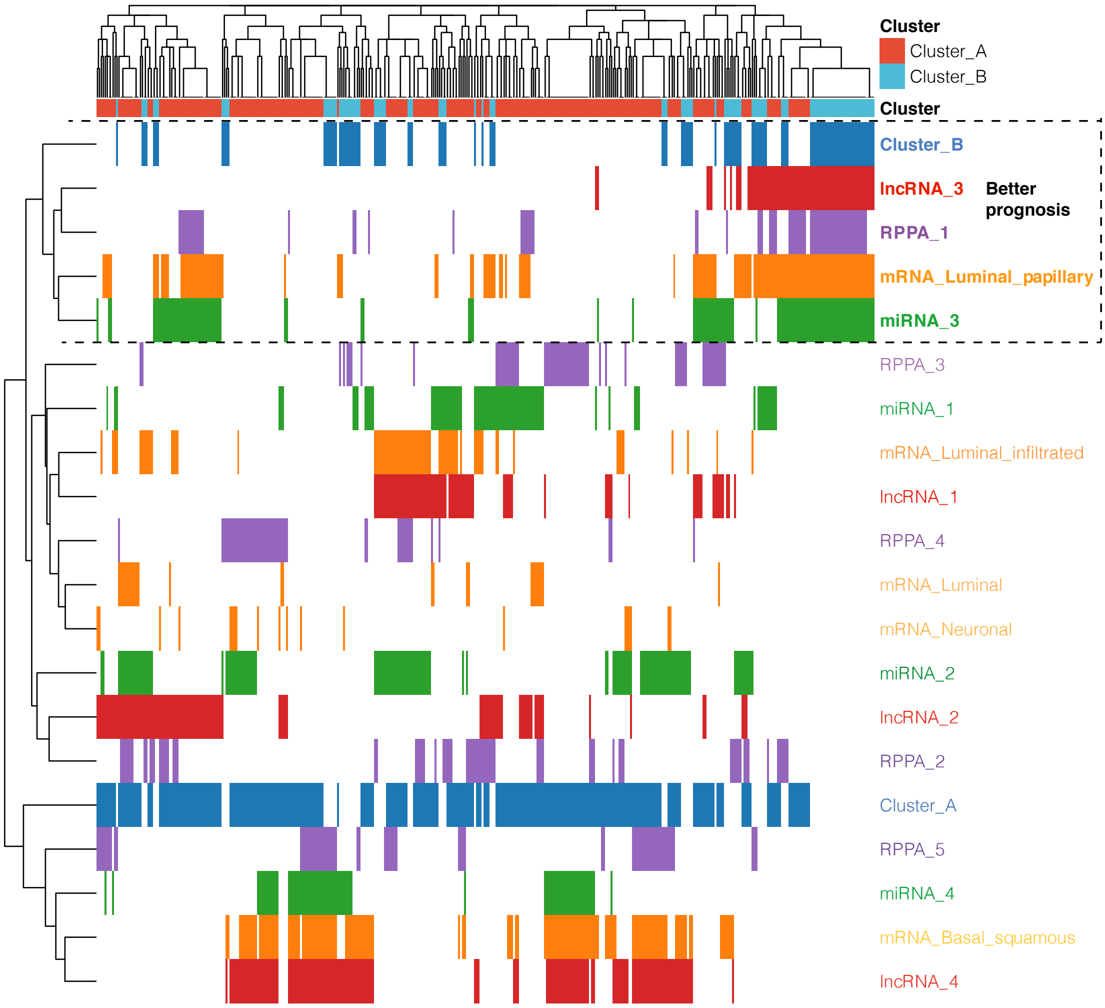

Supplementary Figure 10. Comparison of the two genomic subtypes (Cluster A and B) defined in our study with the molecular subtypes defined by TCGA at different expression levels. Further comparison of these two genomic subtypes with the molecular subtypes defined by TCGA at different expression levels showed that genomic cluster B tended to be clustered with the molecular subtypes mRNA_Luminal_papillary, lncRNA_3, miRNA_3 and RPPA_1 in Dataset 1 patients.

Supplementary Fig 11

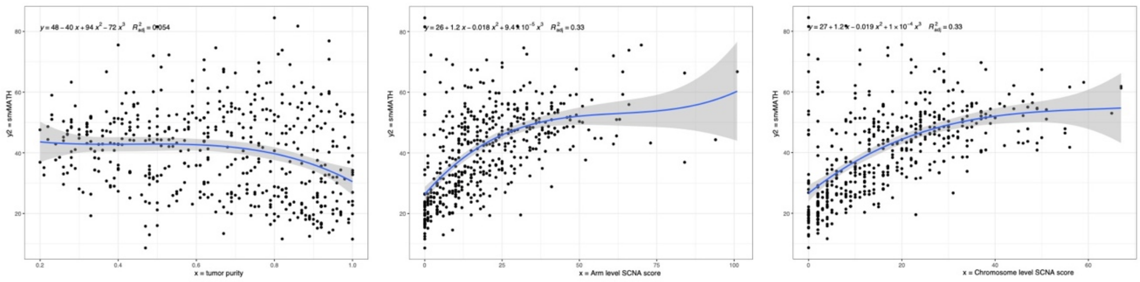

Supplementary Figure 11. The correlation analysis of MATH score with tumor purity, Arm/Chromosome level SCNA score. Each point represents each patient.

Legends for each Supplementary Table files:
Supplementary Table 1. Clinical characteristics of patients used for this study.

Supplementary Table 2. Clinical characteristics associated with the two subtypes of bladder cancer.

Supplementary Table 3. Statistics of Somatic single nucleotide variants (SNVs), Insertions/deletions (Indels) and Somatic copy number alterations (SCNAs).

Supplementary Table 4. Comparison of mutational signatures identified in our study to COSMIC mutational signatures (Related to **Figure 1a**). (a) Cosine similarity. (b) Pearson correlation efficient.

Supplementary Table 5. Mutation table of the *AHNAK/ERCC2/HRAS* genes among Asian/Black/White populations (Related to **Figure 2c**).

Supplementary Table 6. Significantly mutated genes (SMGs) identified by MutSig2CV. SMGs were filtered with $P < 0.05$ & $q < 0.1$. A SMG should express in above 75% of the TCGA-BLCA patients and with at least three read counts. Also, they should be reported as the census of human cancer genes (https://cancer.sanger.ac.uk/census, version: 4_08_47_12_2018.

Supplementary Table 7. Kaplan-Meier survival analysis result of all SMGs and frequent SCNAs events according to their clonal or subclonal vs wild state. Mutant count means the number of patients with this state mutation.

Supplementary Table 8. Predominance of mutation acquisitions in patients with cluster A and B bladder cancer patients. (Mutated frequency and difference test in two subtypes).
