# Supplementary material for: Integrated analysis of racial disparities in genomic architecture identifies a trans‐ancestry prognostic subtype in bladder cancer
Source: Mol Oncol. 2022 Dec 29;17(4):564–81. doi: 10.1002/1878-0261.13360 (PMC10061287; doi:10.1002/1878-0261.13360)
Supplement: Supplementary file 2 — Table S1. Clinical characteristics of patients used for this study. [file MOL2-17-564-s008.doc]

**Supplementary Table 1. Clinical characteristics of patients used for this study**

| **Factor** | **Dataset 1 (TCGA-BLCA)** | **Dataset 2 (Chinese-BLCA)** |  |
| --- | --- | --- | --- |
| **Dataset *Size (Asian, Black, White, Not reported)*** | | | |
|  | 408 (43, 23, 324, 18) | 97 (97, 0, 0, 0) |  |
| ***Histopathological subtype size (Asian, Black, White)*** | | | |
| MIBC | 390 (43, 23, 324) | 60 (60, 0, 0) |  |
| NMIBC | 0 (0, 0, 0) | 37 (37, 0, 0) |  |
| ***Follow up survival information*** | | | |
|  | Yes | Not available |  |
| ***Smoking status (Yes, No)*** | | | |
|  | 389 (180, 209) | Not available |  |
| ***Age subgroup (Asian, Black, White in MIBC) (Asian, Black, White in NMIBC)*** | | | |
| Young | 31 (9, 20, 2) (0, 0, 0) | 28 (14, 0, 0) (14, 0, 0) |  |
| Old | 358 (34, 303, 21) (0, 0, 0) | 69 (46, 0, 0) (23, 0, 0) |  |
| ***Data type (used for analysis)*** | | | |
| Mutation | Yes | Yes |  |
| SCNA | Yes | Yes |  |
| ***Subclusters of clonal architectures (Asian, Black, White)*** | | | |
| Cluster A | 272 (21, 17, 234) | 35 (35, 0, 0) |  |
| Cluster B | 108 (22, 6, 80) | 51 (51, 0, 0) |  |
| ***Gender (Asian, Black, White in MIBC) (Asian, Black, White in NMIBC)*** | | | |
| Male | 290 (35, 242, 13) (0, 0, 0) | 86 (53, 0, 0) (33, 0, 0) |  |
| Female | 99 (8, 81, 10) (0, 0, 0) | 11 (7, 0, 0) (4, 0, 0) |  |
| ***TNM Stage (Asian, Black, White in MIBC) (Asian, Black, White in NMIBC)*** | | | |
| Stage i & Stage ii | 126 (31, 87, 8) (0, 0, 0) | 90 (53, 0, 0) (37, 0, 0) |  |
| Stage iii & Stage iv | 263 (12, 236, 15) (0, 0, 0) | 7 (7, 0, 0) (0, 0, 0) |  |
