# Supplementary material for: Integrated analysis of racial disparities in genomic architecture identifies a trans‐ancestry prognostic subtype in bladder cancer
Source: Mol Oncol. 2022 Dec 29;17(4):564–81. doi: 10.1002/1878-0261.13360 (PMC10061287; doi:10.1002/1878-0261.13360)
Supplement: Supplementary file 3 — Table S2. Clinical characteristics associated with the two subtypes of bladder cancer. [file MOL2-17-564-s004.doc]

**Supplementary Table 2. Clinical characteristics associated with the two subtypes of bladder cancer**

| **Factor** | **Cluster A** | **Cluster B** | **P value** |
| --- | --- | --- | --- |
| ***Dataset Size (MIBC, NMIBC)*** | | | |
| Chinese-BLCA | 35 (27, 8) | 51 (25, 26) | *NMIBC patients enriched in Cluster B ( < .001 )* |
| TCGA-BLCA | 287 (287, 0) | 111 (111, 0) |  |
| ***Follow up survival information*** | | | |
| Chinese-BLCA | 0 | 0 |  |
| TCGA-BLCA | 284 | 110 |  |
| ***Age, median (range)*** | | | |
| Chinese-BLCA | 69 (44 ~ 87) | 62 (28 ~ 84) |  |
| TCGA-BLCA | 71 (35 ~ 91) | 66 (39 ~ 89) |  |
| ***Mutation Signature Cluster (MSig1, MSig2, MSig3, MSig4)*** | | | |
| Chinese-BLCA | 35 (4, 29, 1, 1) | 51 (10, 40, 1, 0) |  |
| TCGA-BLCA | 272 (189, 4, 22, 57) | 107 (82, 2, 9, 14) |  |
| ***Metastatic (No, Yes)*** | | | |
| TCGA-BLCA | 198 (66, 132) | 78 (41, 37) | *non-metastatic patients enriched in Cluster B ( < .001*) |
| ***Whole Genome Doublings Number (0, 1, 2)*** | | | |
| Chinese-BLCA | 35 (11, 17, 7) | 51 (45, 5, 1) |  |
| TCGA-BLCA | 282 (63, 180, 39) | 109 (87, 20, 2) |  |
| ***Racial origins (Asian, Black, White)*** | | | |
| Chinese-BLCA | 35 (35, 0, 0) | 51 (51, 0, 0) |  |
| TCGA-BLCA | 272 (21, 17, 234) | 108 (22, 6, 80) |  |
| ***Gender (female, male)*** | | | |
| Chinese-BLCA | 35 (6, 29) | 51 (3, 48) |  |
| TCGA-BLCA | 282 (78, 208) | 110 (25, 85) |  |
| ***TNM Stage (stage I & stage II, stage III & stage IV)*** | | | |
| Chinese-BLCA | 35 (34, 1) | 51 (48, 3) |  |
| TCGA-BLCA | 286 (84, 202) | 110 (48, 62) |  |
