# Supplementary material for: Integrated analysis of racial disparities in genomic architecture identifies a trans‐ancestry prognostic subtype in bladder cancer
Source: Mol Oncol. 2022 Dec 29;17(4):564–81. doi: 10.1002/1878-0261.13360 (PMC10061287; doi:10.1002/1878-0261.13360)
Supplement: Supplementary file 4 — Table S3. Statistics of somatic single nucleotide variants (SNVs), insertions/deletions (Indels), and somatic copy number alterations (SCNAs). [file MOL2-17-564-s002.doc]

**Supplementary Table 3. Statistics of Somatic single nucleotide variants (SNVs), Insertions/deletions (Indels) and Somatic copy number alterations (SCNAs)**

| **Clonal state** | **SNVs (n = 132, 100)** | **Indels (n = 4, 246)** | **SCNAs (n = 21, 779)** |
| --- | --- | --- | --- |
| Clonal | 117, 321 (88.8%) | 2, 951 (69.5%) | 12, 825 (58.9%) |
| Sub-clonal | 14, 779 (11.2%) | 1, 295 (30.5%) | 8, 954 (41.1%) |
